# A new species of *Psydrax* (Vanguerieae, Rubiaceae) from the Gia Lai Plateau, southern Vietnam

**DOI:** 10.3897/phytokeys.149.51710

**Published:** 2020-06-03

**Authors:** Bui Hong Quang, The Bach Tran, Thi Dung Ha, Hai Do Van, Huong Nguyen Thi Thanh, Ha Bui Thu, Van Son Dang

**Affiliations:** 1 Department of Botany, Institute of Ecology and Biological Resources, Vietnam Academy of Science and Technology, 18 Hoang Quoc Viet, Cau Giay, Ha Noi, Vietnam; 2 Graduate University of Science and Technology, Vietnam Academy of Science and Technology, 18 Hoang Quoc Viet, Cau Giay, Ha Noi, Vietnam; 3 Faculty of Biology, Hanoi National University of Education, 136 Xuan Thuy, Cau Giay, Ha Noi, Vietnam; 4 Institute of Tropical Biology, Vietnam Academy of Science and Technology, 85 Tran Quoc Toan Street, District 3, Ho Chi Minh City, Vietnam

**Keywords:** Gia Lai, new species, *Psydrax*, Rubiaceae, *Vanguerieae*, Vietnam

## Abstract

A new species of Rubiaceae, *Psydraxgialaiensis* B.H.Quang, T.B.Tran & V.S.Dang, **sp. nov.**, is described and illustrated from the Kon Chu Rang Nature Reserve, Gia Lai Province, southern Vietnam. This species is characterized by having strigose branches and leaves, a conspicuously bulging, short corolla tube, an accrescent nectary disc and a style with a dense tuft of hairs, which clearly distinguishes it from the other species in the genus. A description, vernacular name, conservation assessment, illustration, photographs, and a key to the species of *Psydrax* in Vietnam are provided.

## Introduction

The Vanguerieae is a monophyletic tribe of the Rubiaceae characterized by axillary inflorescences, valvate corolla aestivation, a knob-like stylar head, secondary pollen presentation, pendulous ovules, and fleshy fruits with two to ten pyrenes (Lantz andBremer 2004; Razafimandimbison et al. 2009; Kainulainen and Razafimandimbison 2016). Currently, four genera of the tribe *Vanguerieae* are known in Vietnam: *Canthium* with 7 species, *Meyna* with 4 species, *Psydrax* with 3 species and *Vangueria* with 1 species (Pitard 1924; Pham 2000).

*Psydrax* Gaertn. (Vanguerieae, Rubiaceae) includes about 87 species distributed in Africa, Asia, Australasia and the Pacific (Bridson 1985; Lantz and Bremer 2004, 2005; Razafimandimbison et al. 2009; Chen et al. 2011; WCSP 2020). The genus is characterized by having leaves typically subcoriaceous to coriaceous and drying light green or occasionally chartaceous, calyx limb truncate or 4- or 5-dentate, only occasionally equaling the disc but usually much shorter, anthers usually reflexed, fruit obovoid and distinctly bilobed, pyrene cartilaginous to woody with shallow apical crest, endosperm entire (Bridson 1985; Cheek and Sonké 2004; Reynolds and Henderson 2004; Chen et al. 2011; Arriola and Alejandro 2013). In Vietnam, three species of the genus *Psydrax* are recorded: *P.dicoccos* Gaertn., *P.pergracilis* (Bourd.) Ridsdale and *P.umbellatus* (Wight) Bridson (Pitard 1924; Pham 2000).

During a botanical survey of the Gia Lai Province, southern Vietnam in 2017, specimens of the genus *Psydrax* were collected in a primary evergreen forest in the Kon Chu Rang Nature Reserve, at c. 1000 m elevation. After comparing with the specimens in the herbaria HN, MAK, KAG, and VNM, and specimen images on the website of JSTOR Global Plants (https://plants.jstor.org/), and consulting the relevant literature (Pitard 1924; Bridson 1985; Pham 2000; Reynolds and Henderson 2004; Chen et al. 2011), we determined that our specimens represent a new species.

## Materials and methods

All morphological characters of the new species were observed from living and dried specimens; measurements were made using a ruler accurate to 0.5 mm. Herbarium material was stored at the Institute of Ecology and Biological Resources (**HN**) and the Institute of Tropical Biology (**VNM**). The photographs were taken with a Canon 600D fitted with an EF 100 mm f/2.8 Macro USM lens. The conservation status of the new species was assessed according to the guidelines of the International Union for Conservation of Nature (IUCN 2019).

## Taxonomy

### Psydrax gialaiensis

Taxon classificationPlantaeGentianalesRubiaceae

B.H.Quang, T.B.Tran & V.S.Dang sp. nov.

AA7F4CBB-CDDB-5258-A9A1-1B94B9488DAC

urn:lsid:ipni.org:names:77209840-1

Figures 1 , 2 , 3

#### Diagnosis.

Differs from all other known species of *Psydrax* by the strigose pubescence on branches and leaves (vs. glabrous), the conspicuously bulged corolla tube (vs. cylindrical) and the style with a tuft of dense hairs (vs. glabrous).

#### Type.

Vietnam. Gia Lai Province, Kon Chu Rang Nature Reserve, elevation 989 m, 14°30'50.03"N, 108°32'45.01"E, 19 September 2017, *H.Q. Bui*, *V.H. Do*, *T.H. Duong*, *H.S. Doan*, *D.B. Tran KCR 316* (holotype HN!; isotype HN!, VNM!).

**Figure 1.**
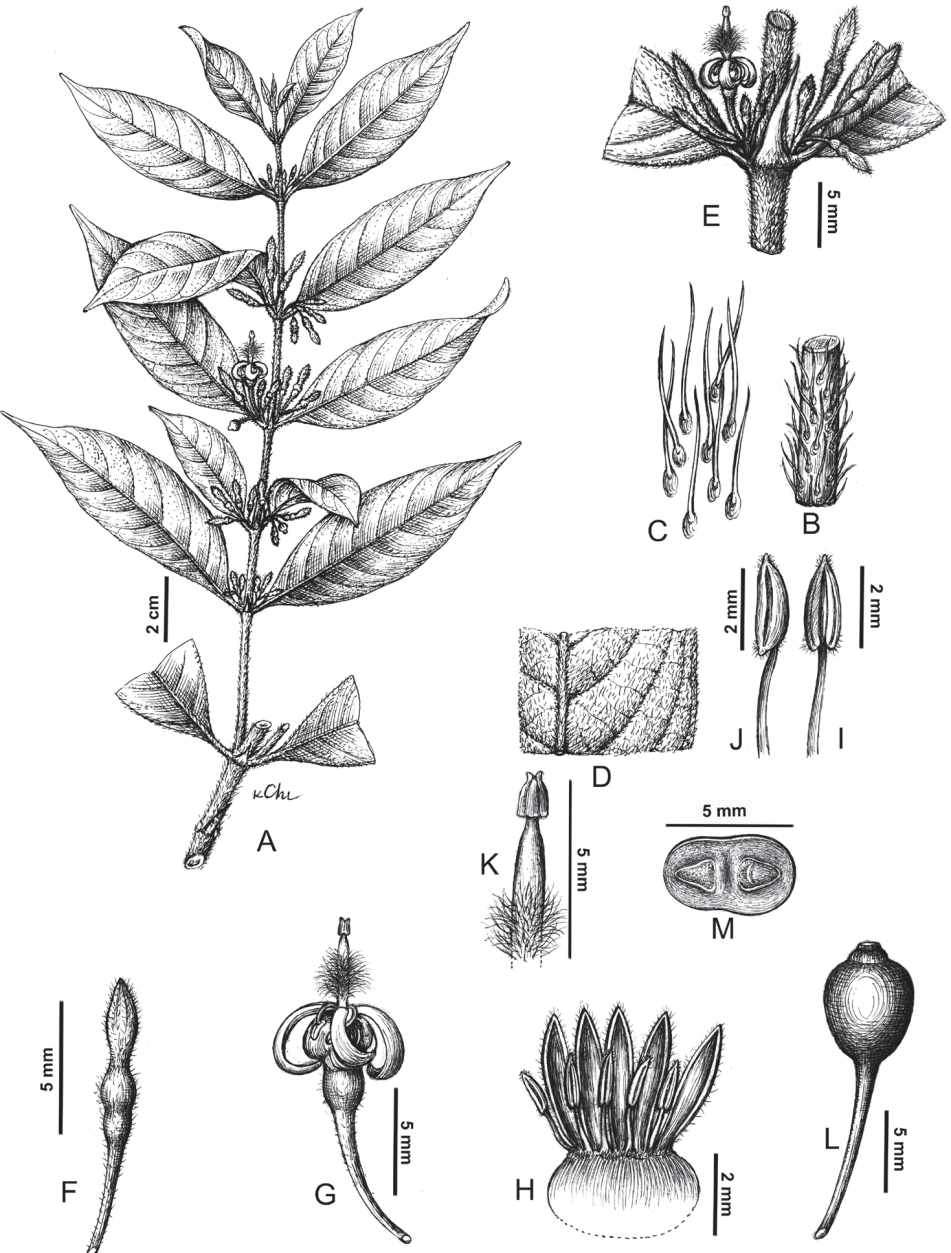
*Psydraxgialaiensis* B.H.Quang, T.B.Tran & V.S.Dang (from the holotype) **A** flowering branch **B** strigose stem **C** hairs **D** adaxial view of a strigose leaf **E** inflorescence and stipule **F** flower bud **G** flower **H** opened flower **I** abaxial view of stamen **J** side view of stamen **K** style and stigma **L** immature fruit **M** cross section of the fruit. Drawn by Le Kim Chi.

#### Description.

Shrubs, 2–3 m tall; branches slender, terete, strigose and greyish-brown when dry. Leaves opposite; petioles 3–5 mm long, strigose; blades oblong or elliptic-oblong, 6–12 × 2–3 cm, coriaceous, strigillose adaxially, strigose abaxially, base cuneate to acute, apex acuminate, acumen 8–12 mm long; secondary veins 5–8 on each side of the midrib, conspicuous abaxially, domatia absent. Stipules with long needle-like awn, 4–6 × 2–3 mm, glabrous abaxially and strigose adaxially; sheath 1–1.3 mm long; awn 1–1.5 mm long. Inflorescences 3–6-flowered, pedunculate; peduncle 0.5–2.5 mm long; bracts small, lanceolate, 0.5–0.8 mm long, strigose. Flowers pedicellate; pedicels 7–10 mm long, sparsely strigillose to sub-glabrous; bracteoles absent; flower bud green, apex acuminate. Calyx dark green, sparsely strigillose; calyx lobes 5, triangular, 0.5–1 mm long. Corolla 8.5–10 mm long, greenish, sparse hairs outside; tube conspicuously bulged, 2–2.5 mm long, with a ring of deflexed white hairs inside; lobes 5, oblong or lanceolate, 6.5–7.5 × 2–3 mm, obtuse or subacute at apex. Stamens 5, reflexed; filaments 2–2.5 mm long; anthers oblong, 2–2.5 mm long, white, pubescent. Disc glabrous, 0.8–1 mm in height in flowers, accrescent in fruit, reaching a height of 1–1.5 mm and becoming white. Ovary cupular, c. 4–4.5 mm long, dark green, sparsely strigillose, bilocular, 1–ovule per locule; style and stigma white, style 7.5–8 mm long, exceeding corolla tube for 6–6.5 mm, the middle part of the style with a dense tuft of white hairs; stigma 1 × 0.6 mm, bifid, revealing a cleft at the apex and a slight basal recess. Fruit obovoid, strongly bilobed, 5–6 × 3–4 mm, green to blue green, sparsely strigose, pyrenes 2, cartilaginous. Mature seeds unknown, slightly ellipsoid to oblong, at least 2–3 × 1–2 mm; embryo with cotyledons small, set parallel to the ventral face of the seed.

**Figure 2.**
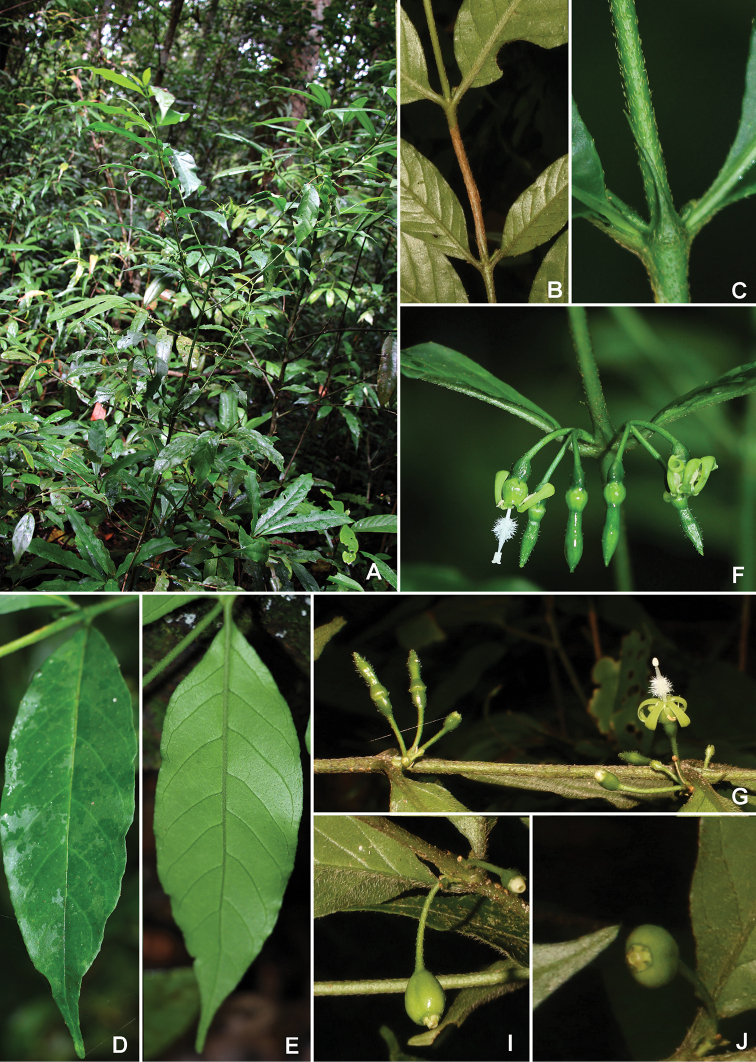
*Psydraxgialaiensis* B.H.Quang, T.B.Tran & V.S.Dang **A** habit **B** branch **C** stipule **D** adaxial leaf surface **E** abaxial leaf surface **F** inflorescences **G** flowering branch **I–J** fruits (photos by H.Q.Bui & D.V.Hai).

#### Other specimen examined.

Vietnam. Gia Lai Province, Kon Chu Rang Nature Reserve, elevation 1000 m, 14°30'50.3"N, 108°32'45.1"E, 20 September 2017, *H.Q. Bui*, *V.H. Do*, *T.H. Duong*, *H.S. Doan*, *D.B. Tran KCR 073* (HN!, VNM).

#### Phenology.

Flowering and fruiting specimens were collected in September.

#### Distribution and habitat.

*Psydraxgialaiensis* is only known from the Kon Chu Rang Nature Reserve, Gia Lai Province, southern Vietnam. It grows in primary evergreen forests, where *Pavettabauchei* Bremek., *Lasianthuscurtisii* King & Gamble, *Popowiapisocarpa* (Blume) Endl. ex Walp., *Fissistigmataynguyenense* Bân, *Ardisiaverbascifolia* Mez and *Litseaclemensii* C.K.Allen are dominant.

**Figure 3.**
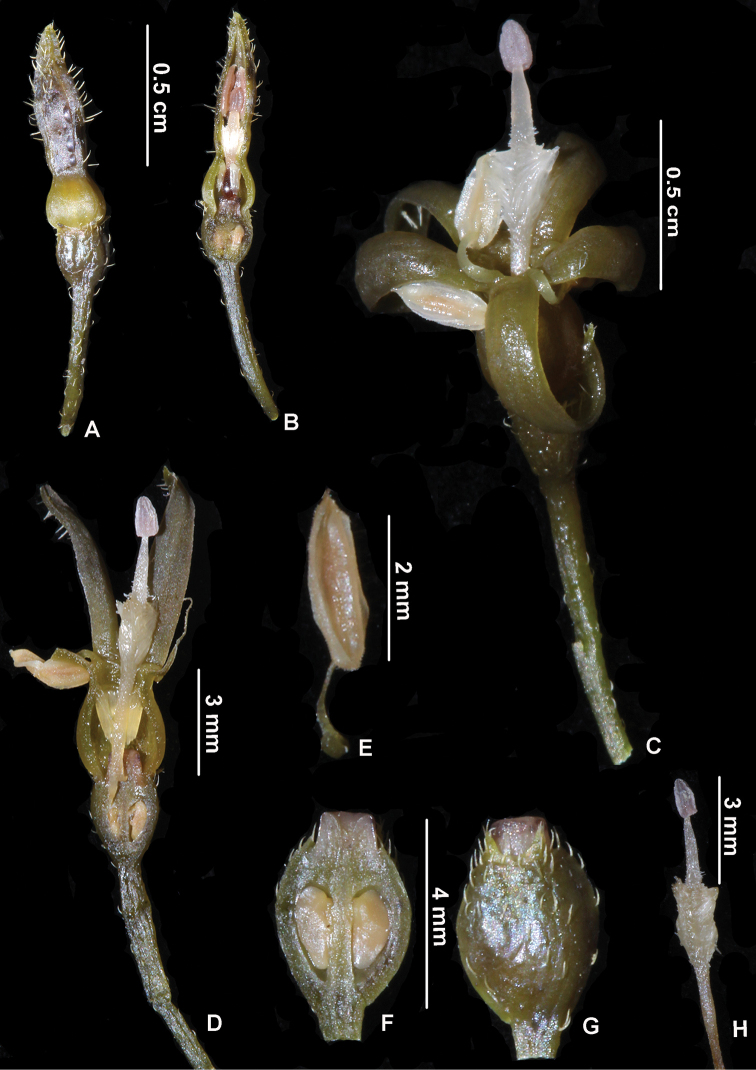
*Psydraxgialaiensis* B.H.Quang, T.B.Tran & V.S.Dang **A, B** flower bud **C, D** flower **E** filament and anther **F** longitudinal section through an ovary showing the 2 locules **G** ovary **H** style with a dense tuft of hairs and stigma (photos by H.Q.Bui, T.D.Binh & V.A.Thuong).

#### Etymology.

The specific epithet refers to the type locality, the Gia Lai Province in Vietnam.

#### Vernacular name.

Căng gia lai

#### Conservation status.

*Psydraxgialaiensis* is commonly found in primary evergreen forests in the Kon Chu Rang Nature Reserve where it is well protected. Therefore, we suggest a status of Least Concern (LC) according to the IUCN Red List Categories (IUCN 2019).

#### Note.

*Psydraxgialaiensis* is distinct from the other previously known species of *Psydrax* in Vietnam based on several characters mentioned in Table 1.

**Table 1.** Morphological comparison of *Psydraxgialaiensis* with the other Vietnamese species (modified from Pitard 1924; Pham 2000; Chen et al. 2011).

Characters	*P.gialaiensis*	*P.dicoccos*	*P.pergracilis*	*P.umbellatus*
Hairiness of branches	strigose	glabrous	glabrous	glabrous
Leaf size (cm)	6–12 × 2–3	4–10 × 1.5–4	5–10 × 1.5–5	8–17 × 4–8
Number of secondary veins	5–8	3–5	3–4	6–8
Petiole length (mm)	3–5	6–15	4–8	5–8
Stipule length (mm)	4–6	3–5	5–7	8–10
Number of flowers per infloresscence	3–6	-	-	-
Peduncle length (mm)	0.5–2.5	3–8	6–25	6–10
Bract length (mm)	0.5–0.8	reduced	-	3
Pedicel length (mm)	7–10	3–8	-	4–6
Calyx lobe length (mm)	0.5–1	0.5	0.5	0.5
Corolla tube length (mm)	2–2.5	3	10	2
Corolla lobe length (mm)	6.5–7.5	2.5–3	2	2
Style	with a dense tuft of hairs	glabrous	glabrous	glabrous
Fruit size (mm)	5–6 × 3–4	8–10 × 6–8	6–14 × 5–10	6–8 × 4–7

## Discussion

*Psydrax* is distinguished from the other genera of tribe Vanguerieae represented in Vietnam by having leaves typically subcoriaceous to coriaceous, calyx limb truncate or 4- or 5-dentate, anthers completely exserted and usually reflexed, fruit obovoid and distinctly bilobed, as well as pyrene cartilaginous to woody with shallow apical crest (Bridson 1985; Cheek and Sonké 2004; Reynolds and Henderson 2004; Chen et al. 2011; Arriola and Alejandro 2013). The new species we collected in the Kon Chu Rang Nature Reserve is shown to be a member of the genus *Psydrax* because it is characterized by these features. However, it is unlike other species of *Psydrax*, which are usually entirely glabrous, the exception being *P.maingayi* (Hook.f.) Bridson and allied taxa (Malaya/Indonesia). This species has leaves that are finely pubescent beneath and straight-sided corolla tubes, whereas in *P.gialaiensis* the pubescence is strigose and the corolla tube conspicuously bulging. Especially, the new species is distinct in its style with a tuft of dense hairs, its accrescent disc and its pubescent anthers. Molecular phylogenetic studies on the new species are needed.

With three species of *Psydrax* reported in Vietnam, *P.gialaiensis* is the only species of the genus known from Kon Chu Rang Nature Reserve, Gia Lai Province. Unlike the habitat of the other three species, which occur in the secondary evergreen forest, evergreen mixed forest and along the edge of forests, *P.gialaiensis* is found only in primary evergreen forests.

### Key to the species of *Psydrax* in Vietnam

**Table:** 

1	Branches and leaves strigose; style with a dense tuft of hairs	***P.gialaiensis***
–	Branches and leaves glabrous; style glabrous	**2**
2	Secondary veins 6–8 pairs; stipules ≥ 8 mm long	***P.umbellatus***
–	Secondary veins 3–5 pairs; stipules ≤ 7 mm long	**3**
3	Leaves oblong-lanceolate; calyx tube 1.5 mm long; corolla tube 10 mm long	***P.pergracilis***
–	Leaves ovate, elliptic, obovate, ovate-elliptic, or ovate-lanceolate; calyx tube 1–1.2 mm long; corolla tube 3 mm long	***P.dicoccos***

## Supplementary Material

XML Treatment for Psydrax gialaiensis
